# Fulminant hemolysis in glucose‐6‐phosphate dehydrogenase deficiency

**DOI:** 10.1002/ccr3.1290

**Published:** 2017-11-24

**Authors:** Bushra Moiz, Sidra Asad Ali

**Affiliations:** ^1^ Section of Hematology and Transfusion Medicine Pathology and Laboratory Medicine The Aga Khan University Karachi Pakistan; ^2^ Junior Consultant Hematology Patel Hospital Karachi Pakistan

**Keywords:** Glucose‐6‐phosphate dehydrogenase deficiency, hemolysis, viral hepatitis, X‐linked inherited disorder

## Abstract

Glucose‐6‐phosphate dehydrogenase (G6PD) deficiency is an X‐linked disorder affecting some 400 million people worldwide. Though clinically silent, it may result in hemolysis on oxidative stress induced by drugs or infections. Viral hepatitis A with coexisting G6PD deficiency can be devastating associated with severe hemolysis, anemia, renal failure, and hepatic encephalopathy.

## Introduction

Glucose‐6‐phosphate dehydrogenase (G6PD) is a pentose pathway enzyme which catalyzes the rate‐limiting step in the reduction of nicotine amide adenine dinucleotide phosphate (NADP) to NADPH. The presence of NADPH is critical in producing reduced glutathione required for eradicating free radicals from the cells. G6PD‐deficient red cells undergo hemolysis when challenged with certain foods, medications, and infections as they are unable to handle the excessive oxygen‐derived free radicals under these circumstances. Though most G6PD‐deficient individuals are asymptomatic, they are at risk of hemolysis and consequential hyperbilirubinemia on oxidative stress. G6PD deficiency is the most common of all genetic deficiencies affecting over 400 million people in the world. It is quite frequent in Pakistan with a prevalence of 2–8%. Here, we report a case of a young girl who presented with complicated hepatitis A due to underlying G6PD deficiency.

## Case Report

**Figure 1:**
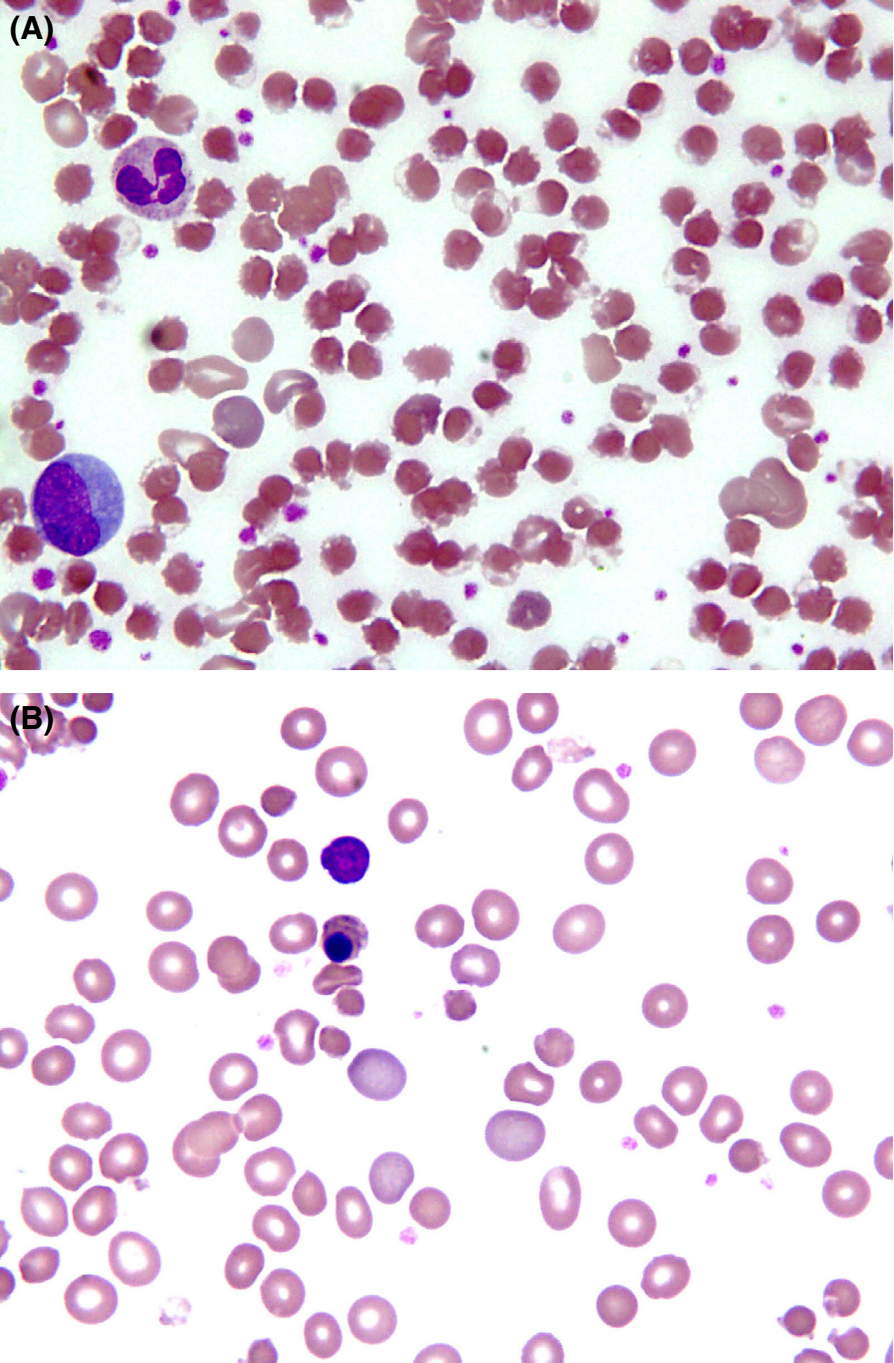
(A) Severe hemolysis on day of presentation marked by blister cells. (B) Second day of hospital admission showing reactive polychromasia and nucleated red cells.

A 10–year‐old girl presented with drowsiness for 3 days. She had history of fever, abdominal pain, and dyspnea since few days. She was febrile, jaundiced, and arousable. Her hemoglobin was 87 g/dL, MCV 82.1 fL, MCH 28.6 pg, white cells 53.5 × 10^9^/L, and platelets 266 × 10^9^/L. Blood film [see photograph] revealed polychromasia, nucleated red blood cells, and numerous blister cells. Reticulocyte count was 25%, and total bilirubin 45.8 mg/dL with direct component of 22.5 mg/dL, serum glutamic‐pyruvic transaminase (SGPT) of 4329 IU/L, prothrombin time (PT) 15.8 sec, and serum creatinine 0.7 mg/dL and anti‐Hepatitis A IgM antibodies were reactive. The clinical diagnosis was hepatic encephalopathy secondary to fulminant hepatitis A. The presence of blister cells in peripheral film on day of presentation (Fig. 1A) prompted glucose‐6‐phosphate dehydrogenase assay which was quantified as 0.5 U/gHb consistent with severe G6PD deficiency. Her peripheral film done next day showed a reduction in blister cells, increase in nucleated and polychromatic red cells (Fig. 1B). Supportive care was initiated, and she was discharged from hospital after a fortnight at bilirubin of 5.7 mg/dL and hemoglobin of 10 g/dL without any complications.

## Discussion

Viral hepatitis A is a water‐borne infection that is usually self‐limiting. The disease may cause severe hemolysis due to underlying G6PD deficiency leading to fulminant hepatic failure. Few cases of such complicated hepatitis have been described due to hepatitis B and hepatitis E 1.

Our patient had severe manifestation of hepatitis A because of underlying G6PD deficiency while acute hemolysis was precipitated by infection. A handful of cases with interaction of hepatitis A and G6PD deficiency were described in the literature. The possible explanation was G6PD‐deficient hepatocytes with reduced glutathione were unable to withstand viral insult cumulating free radicals resulting in delayed hepatocytes repair 2.

Several reports have shown increased morbidity and fulminant clinical course of acute viral hepatitis when develop with underlying G6PD deficiency. Jain et al. studied 10 patients with viral hepatitis A and E who were G6PD deficient and found significantly high levels of indirect bilirubin and prothrombin time in these individuals as compared to control group 3. Cases of acute renal failure leading to hemodialysis secondary to severe intravascular hemolysis in viral hepatitis with preexisting G6PD deficiency have also been reported 4.

Our patient developed severe hemolysis leading to protracted hospital stay but recovered successfully through supportive management.

## Conflict of Interest

None declared.

## Authorship

BM: diagnosed the case and wrote the manuscript. SA: took photographs and history of the patient and wrote the manuscript.

## References

[1] Karki, P. , S. Malik , B. Mallick , V. Sharma , and S. S. Rana . 2016 Massive Hemolysis causing renal failure in acute hepatitis E infection. J. Clin. Transl. Hepatol. 4:345–347. 28097104 10.14218/JCTH.2016.00042 PMC5225155

[2] Dea, Bandyopadhyay . 2015 Severe hemolysis as the first manifestation of acute hepatitis A in an adult with G6PD deficiency and positive ANA. Am. J. Med. Case Rep. 3:158–159.

[3] Jain, A. K. , S. Sircar , M. Jain , S. Adkar , C. Waghmare , and F. Chahwala . 2013 Increased morbidity in acute viral hepatitis with glucose‐6‐phosphate dehydrogenase deficiency. Indian J. Gastroenterol. 2:133–134. 10.1007/s12664-012-0226-9 22869048

[4] Abid, S. , and A. H. Khan . 2002 Severe hemolysis and renal failure in glucose‐6‐phosphate dehydrogenase deficient patients with hepatitis E. Am. J. Gastroenterol. 97:1544–1547. 12094881 10.1111/j.1572-0241.2002.05740.x

